# Expanded detection of canine enteric viruses in UK dogs with diarrhoea

**DOI:** 10.1099/mgen.0.001799

**Published:** 2026-09-08

**Authors:** Jack Pilgrim, Aleksandra Rzeszutek, Edward Cunningham-Oakes, Shirley Bonner, Larry Roberts, Alistair C Darby, Alan D Radford

**Affiliations:** 1 Institute of Infection, Veterinary and Ecological Sciences, Faculty of Health and Life Sciences, University of Liverpool, Liverpool, L69 3BX, UK; 2 School of Food Science and Nutrition, Faculty of Environment, University of Leeds, Leeds LS2 9JT, UK; 3 IDEXX Laboratories, Grange House, Sandbeck Way, Wetherby LS22 7DN, UK; 4 Centre for Genomic Research, Institute of Systems, Molecular and Integrative Biology, Faculty of Health and Life Sciences, University of Liverpool, Liverpool, L69 7ZB, UK

**Keywords:** diarrhoea, dog, high-throughput sequencing, surveillance, UK, viral metagenomics

## Abstract

Canine enteric viruses are an important cause of gastrointestinal disease in pet dogs worldwide. Routine diagnosis often relies on pathogen-specific PCR assays, which may fail to detect some viruses, particularly neglected pathogens or genetically divergent variants of established threats. This limits both clinical characterization of affected patients and broader understanding of disease ecology. To address these limitations, we applied metagenomics and a viral discovery bioinformatics pipeline to faecal samples from diarrhoeic dogs in the UK that had been submitted routinely for PCR-based diagnostic testing. Across 80 dogs, we identified 12 viruses known to infect canids, 9 of which have not previously been reported in UK dogs. Among these, several taxa with prior associations to gastrointestinal disease were identified, including canine sapovirus and canine minute virus. By contrast, for other viruses newly detected in the UK, including bufavirus and rotavirus C, clinical relevance in dogs remains unclear. Notably, an identified protoparvovirus fell within the same species as human–canine-associated parvovirus 1, a recently described lineage detected in both canine and human oropharyngeal samples. We also identified a canine parvovirus 2 strain that clustered with a predominantly wildlife-associated lineage, consistent with occasional exposure at the domestic–wildlife interface rather than established circulation in dogs. These two detections illustrate how genome-level surveillance can help prioritize viruses for targeted investigation of host range and transmission context. Overall, these data broaden the catalogue of viruses associated with diarrhoeic dogs in the UK and support periodic review of diagnostic targets informed by viral metagenomic surveillance, while highlighting the need for controlled studies to assess causality and clinical relevance.

**Impact Statement**

Routine molecular diagnostics for canine diarrhoea are typically limited to predefined pathogens and may therefore overlook genetically divergent, neglected or emerging viruses circulating in companion animal populations. In this study, viral metagenomic sequencing identified several enteric viruses in UK dogs with diarrhoea that were not represented on a routine PCR diagnostic panel, including viruses previously linked to gastrointestinal disease in dogs, as well as poorly characterized taxa requiring further investigation. These findings demonstrate the value of untargeted viral genomics for identifying diagnostic blind spots, generating baseline surveillance data and prioritizing viruses for future epidemiological and functional study. More broadly, this work highlights how periodic integration of viral metagenomic surveillance into veterinary diagnostic frameworks could inform assay design and strengthen detection of previously under-recognized canine viruses.

## Data Summary

All raw sequencing reads and assembled viral genomes generated in this study are archived in the European Nucleotide Archive (ENA) under BioProject accession PRJEB75387. Additional per-sample metadata, diagnostic PCR results and metagenomic virus detections are provided in Table S1. Virus sequences and raw reads are available at the ENA under project accession number PRJEB75387.

## Introduction

Canine diarrhoea is among the most frequent clinical symptoms encountered in small-animal practice and remains a common reason for diagnostic investigation and antimicrobial prescribing [1]. Aetiology of diarrhoea spans dietary and inflammatory causes, as well as infectious agents, including viruses, bacteria and parasites [2]. Although several viral enteropathogens are recognized in dogs, disease expression ranges widely and can be influenced by co-infections and host-related factors that shape the likelihood of clinical signs [3, 4]. Many ill dogs test negative for known pathogens on conventional assays, suggesting that new or variant viruses are likely [5]. A robust understanding of which enteric viruses are circulating locally is therefore relevant both to case investigation and to population-level veterinary surveillance.

Routine diagnostic work-up for diarrhoeic dogs is typically centred on targeted approaches, including culture-based assays for selected bacterial enteropathogens (e.g. *Salmonella* spp. and *Campylobacter* spp.) and commercial PCR panels restricted to predefined viral and bacterial targets [1, 6]. These approaches are valuable for rapid first-line testing, but they are intrinsically limited to expected agents and to the genomic regions targeted by PCR primers. As a result, viruses that are not represented on panels, or that are sufficiently distinct from reference lineages used during assay development, may be under-detected. In addition, for many canine enteric viruses, UK genomic data remain sparse relative to the diversity described internationally, limiting the ability to interpret introductions, lineage turnover and the potential for emergence of divergent variants.

Viral metagenomic sequencing provides a complementary framework for enteric virus surveillance by enabling unbiased and broad detection of viruses in clinical material while also generating genome-scale data for phylogenetic and variant-level analyses, as well as informing periodic refinement of PCR assay design [7]. Metagenomic investigations of canine enteric disease in other settings have expanded the recognized diversity of viruses detected in dogs [8–11], but comparable studies from UK clinical submissions remain limited.

Here, we applied viral metagenomics to a cohort of diarrhoeic canine faecal samples to (i) characterize the spectrum of viruses detected in clinical submissions, (ii) recover genomes to enable phylogenetic placement of key detected taxa and (iii) identify viruses not represented in routine diagnostic panels that merit targeted follow-up. Collectively, these data provide a baseline genomic resource for UK canine enteric virus surveillance and inform the design of subsequent epidemiological studies aimed at resolving disease association and transmission interfaces.

## Methods

### Sample collection

Faecal samples were obtained through the Small Animal Veterinary Surveillance Network virtual biobank, which enables expedited retrieval of surplus clinical specimens from diagnostic laboratories after completion of diagnostic testing and prior to disposal. Samples were received in February 2022 from the biobank and stored at −80 °C until further processing. For this project, residual faecal samples were obtained from 80 dogs presenting with diarrhoea that had previously been screened using the Canine Diarrhoea PCR Panel (CDPP; IDEXX Laboratories), which tests for canine parvovirus-2, canine enteric coronavirus (CECoV), canine distemper virus, *Giardia* spp., *Cryptosporidium* spp., *Clostridium perfringens* and *Salmonella* spp. Although all samples had reportedly undergone CDPP testing, associated diagnostic records could only be retrieved for 74 samples. Samples were selected to include the following: (i) samples in which no pathogen was detected by the CDPP panel, to assess whether additional agents may have been present (*n*=18); (ii) a small subset that had previously undergone CECoV RT-PCR screening targeting the membrane (M) gene in a previous study (*n*=12) [12] was included as external controls; and (iii) samples with single or mixed infections identified by the CDPP panel, to investigate whether other potential infectious causes may also have been present (*n*=44). Where available, accompanying metadata, including sex, breed, sampling date and postcode, were collated for downstream comparative analyses (Table S1, available in the online Supplementary Material).

### Viral enrichment, nucleic acid extraction and amplification

Faecal samples were processed for viral enrichment, nucleic acid extraction and sequence-independent amplification as described previously [13], with minor modifications. Briefly, 10% faecal suspensions in PBS were clarified by centrifugation, sequentially filtered through 0.8 µm and 0.45 µm membranes, before treatment with Baseline-ZERO DNase (Cambio), TURBO DNase (Thermo Fisher Scientific/Ambion) and RNase ONE Ribonuclease (Promega), to remove unprotected nucleic acids prior to extraction. Total RNA and DNA were extracted using the QIAamp Viral RNA Mini Kit (Qiagen) without carrier RNA or the use of DNase I before performing sequence-independent single-primer amplification (SISPA) [14], with AMPure XP (Beckman Coulter) bead clean-up between stages using a 1.8× bead-to-sample ratio. PBS-only negative controls were used alongside the samples.

### Library preparation and Illumina sequencing

Sequencing libraries were prepared from amplified material using the NEBNext Ultra II FS DNA Library Prep Kit (New England Biolabs) with dual indexing (NEBNext Multiplex Oligos for Illumina). Libraries were sequenced on an Illumina MiSeq platform using 2×250 bp paired-end chemistry, generating a total of 20.6 million reads.

### Read processing and assembly

Adapter and SISPA primer sequences were removed from raw reads using Cutadapt v4.5 [15], followed by quality trimming (minimum sliding-window quality score of 20). *De novo* assembly was performed using MEGAHIT v1.2.9 [16] with default parameters, and contigs shorter than 1,000 nt were excluded from downstream analyses.

### Viral contig identification and curation

To provide an initial overview of viral content, reads were profiled using Kraken2 v2.1.2 [17] against the viral RefSeq database (downloaded on 24 April 2025), and the proportion of viral reads per library was assessed. For contig-level virus discovery, putative viral sequences were identified using complementary homology- and signature-based approaches. Contigs were queried against the Virus-Host DB protein database [18] (downloaded on 8 November 2024) using BLAST+ v2.15.0 [19] with an e-value threshold of 1×10⁻⁵ and minimum query coverage per high-scoring segment of 30%. In parallel, VirSorter2 v2.2.3 [20] was used with default parameters to detect viral contigs; contigs identified by either method were retained for downstream refinement.

Candidate viral contigs were extended using Contig Overlap Based Re-Assembly [21]. Coding sequences were predicted using Prodigal v2.6.3 [22] in metagenomic mode, and translated proteins were screened against RVDB-prot v29.0 [23] using HMMER v3.3.2 [24]; contigs encoding proteins consistent with recognized viral families (e-value ≤1×10⁻⁵) were retained. Genome completeness was assessed using ViralQC [25], retaining contigs with estimated completeness ≥50%. Contigs not scored by ViralQC were evaluated using a secondary classification approach based on MMseqs2 v14.7 [26] searches against an International Committee on Taxonomy of Viruses (ICTV)-derived protein database and a lowest common ancestor assignment framework, applying the same completeness threshold. The resulting high-confidence contig set was dereplicated using dRep v3.4.0 [27] (primary clustering 90% ANI; secondary clustering 95% ANI), and only genomes containing at least one complete open reading frame were retained.

Provisional contig annotations were generated using BLASTx against the Virus-Host DB (e-value ≤1×10⁻⁵; minimum query coverage 30%) to guide downstream phylogenetic placement. Reads were mapped to assembled contigs for all retained genomes using bwa-mem2 v2.2.1 [28] to assess read support and coverage across assemblies. Where genomes were incomplete, reads were additionally mapped to the closest available reference genome sharing ≥90% nucleotide identity to support scaffold-guided extension and local gap filling. Read alignments were processed using the mpileup function implemented in SAMtools [29] to assess breadth of coverage and refine genome sequences. Final assemblies were inspected manually in IGV v2.12.3 [30].

### Phylogenetic analysis and taxonomic assignment

Phylogenetic inference was conducted on dereplicated contigs representing either complete genomes (*Circoviridae*) or complete marker protein sequences from key taxa: *Parvoviridae* NS1, kobuvirus polyprotein, *Astroviridae* capsid, CECoV RdRp, sapovirus polyprotein and rotavirus VP2. Marker open reading frames were compared against the NCBI nr database using BLASTp, with closely related sequences retrieved alongside representative ICTV reference taxa for each group. For marker genes with documented recombination within particular viral taxa (e.g. astrovirus capsid protein), recombination screening was performed using RDP5 [31]. Recombination events were evaluated using multiple detection methods implemented (RDP, GENECONV, MaxChi, Chimaera, SiScan and 3Seq), with multiple-testing correction applied. Sequences were classified as recombinant only when supported by at least four independent methods under a corrected significance threshold (*P*<0.05). Sequences identified as recombinant under these criteria were excluded from downstream phylogenetic analysis. Phylogenetic tree reconstruction was performed as described previously [32]. Briefly, sequences were aligned using MAFFT v7.047 [33], trimmed to remove poorly aligned regions, and maximum-likelihood phylogenies were inferred using IQ-TREE v1.6.12 [34] with ultrafast bootstrap support. Trees were subsequently visualized in RStudio v2024.09.0 [35] using the ggtree package [36].

### Targeted comparative analysis

For selected viruses exhibiting atypical divergence patterns or features of interest, similarity plots were generated in RStudio [35] using a custom sliding-window nucleotide identity script (Supplementary Results) applied to MAFFT [33] alignments (200 nt window; 20 nt steps). Plots were produced using ggplot2 [37]. For Protoparvovirus Dog/89740/UK/2022, a 3D structural model of the core capsid region (VP2) was generated using AlphaFold 3 [38], and key amino-acid differences relative to the closest reference sequence were mapped onto the model.

### Viral abundance, distribution and co-occurrence analyses

Viral abundance was estimated by mapping reads from all libraries to the final dereplicated viral contig set using bwa-mem2 v2.2.1 [28] and summarizing coverage and read counts with CoverM v0.7.0 [39]. A contig was considered present in a library if ≥50% of its length was covered by mapped reads, and contig read counts were aggregated to generate abundance matrices for visualization using pheatmap [40]. To assess the sensitivity of this threshold, supplementary analyses were also performed using relaxed breadth-of-coverage criteria of ≥20% and ≥10% of contig length covered by mapped reads (Table S2). Geographic distributions of viral detections were mapped in R using postcodes for the veterinary practice that submitted the original sample through the postcodes.io API (https://postcodes.io/docs/api/). Maps were generated using Natural Earth UK basemap polygons (via rnaturalearth) and plotted with ggplot2 [37].

## Results

### Summary of viral metagenomic sequencing

**Fig. 1.**
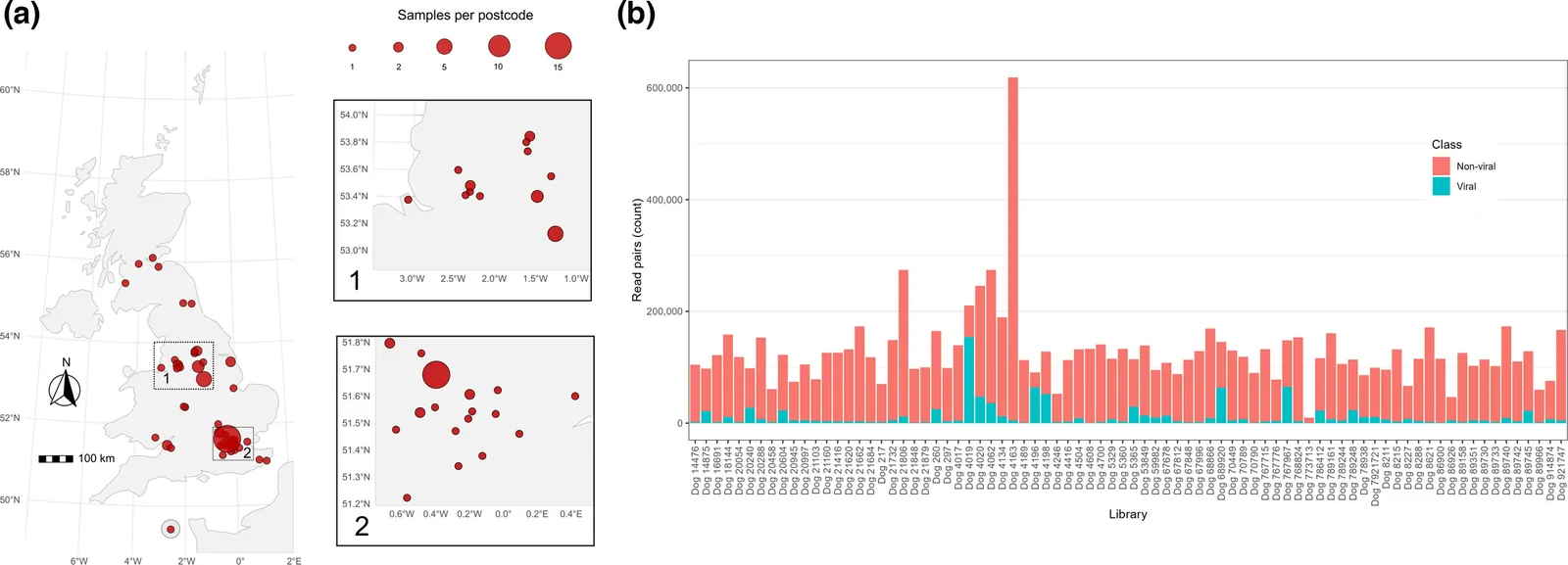
(a) Geographic distribution of collected canine faecal samples. (b) The number of reads in each sample and the proportion identified as viral, as identified by Kraken2.

**Fig. 2.**
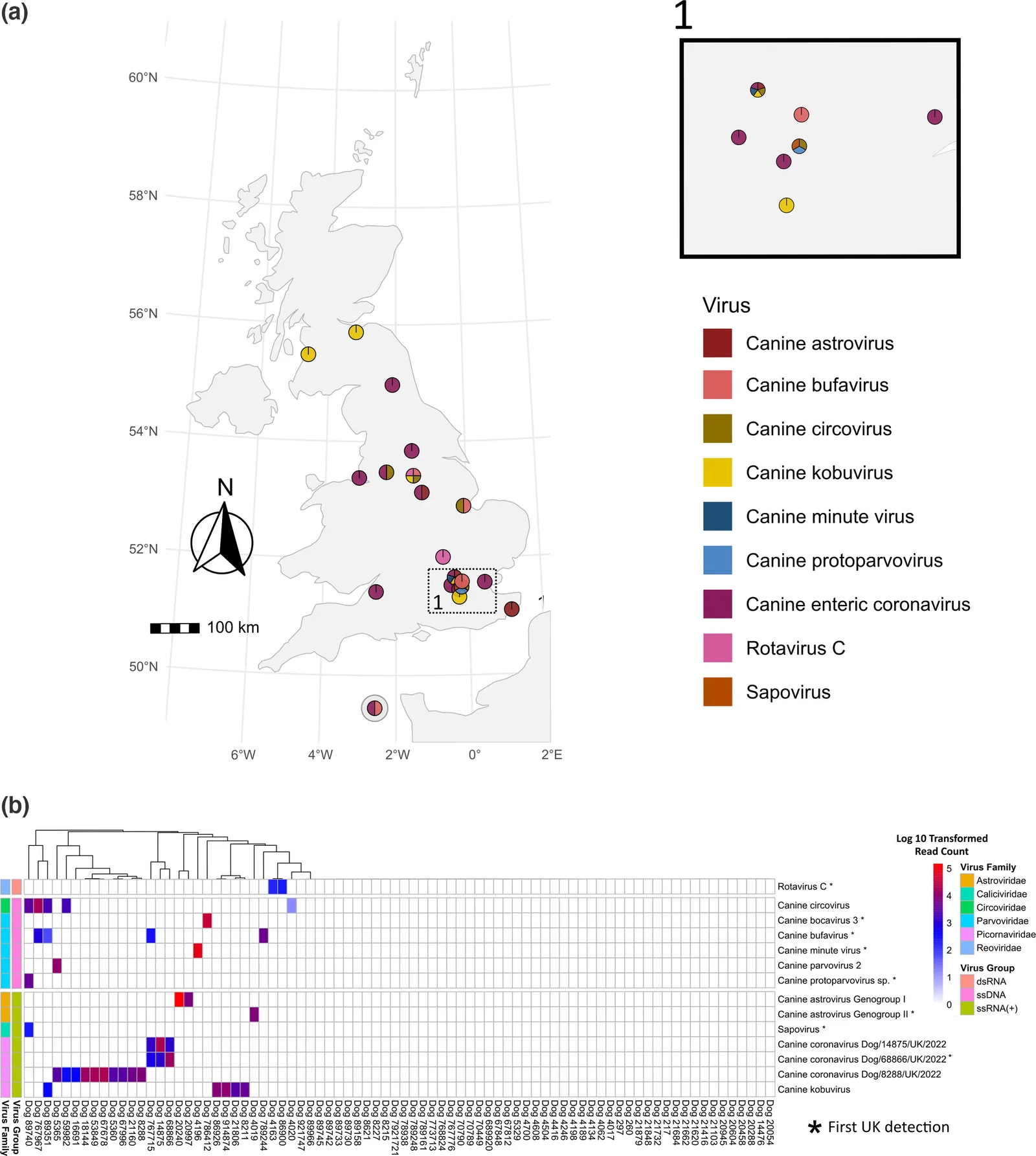
(a) UK distribution of putative canine enteric viruses. Canine bocavirus 3 and canine parvovirus 2 detections are not mapped due to lack of available metadata. (b) Heatmap showing viruses detected in samples and respective read counts.

Viral metagenomic sequencing of 80 canine faecal samples yielded (near-) complete viral genomes in 27 out of 80 samples (33.8%). Across all 80 libraries, sequencing generated a mean of 128,646 read pairs per sample (median 117,888; IQR 98,787–140,619), of which a mean of 12,161 read pairs (9.19%) were classified as viral by Kraken2 (median viral fraction 4.08%; IQR 1.91–10.4%; Fig. 1, Table S3). Of virus-positive samples, 7 out of 27 (25.9%) contained more than 1 virus. After dereplication to collapse redundant near-identical sequences, 14 high-confidence viral genomes were retained, representing 12 species across 7 families, comprising both RNA and DNA viruses (Fig. 2, Table 1).

**Table 1.** Viral genomes recovered from faecal samples. Mean depth coverage refers to dereplicated genomes

Virus	Taxonomy (family; genus)	Detections (n)	GenBank accession IDs	Length (nt)	Mean coverage depth (×)
Canine enteric coronavirus	*Coronaviridae*; *Alphacoronavirus*	13	OZ453977.1, OZ453979.1, OZ453836.1	28,092–29,139	55–125
Canine circovirus	*Circoviridae; Circovirus*	5	OZ453982.1	2,063	305
Canine kobuvirus	*Picornaviridae; Kobuvirus*	5	OZ453980.1	8,012	216
Canine bufavirus	*Parvoviridae; Protoparvovirus*	4	OZ453981.1	4,515	205
Canine astrovirus genogroup I	*Astroviridae; Mamastrovirus*	2	OZ453984.1	6,554	4,330
Canine astrovirus genogroup II	*Astroviridae; Mamastrovirus*	1	OZ453978.1	6,345	225
Rotavirus C	*Reoviridae; Rotavirus*	2	OZ454010.1- OZ454020.1	13,059	6
Sapovirus	*Caliciviridae; Sapovirus*	1	OZ454013.1	2,895	64
Canine minute virus (*Carnivore bocaparvovirus* 1)	*Parvoviridae; Bocaparvovirus*	1	OZ453835.1	5,163	2,538
Canine bocavirus 3	*Parvoviridae; Bocaparvovirus*	1	OZ453983.1	5,165	1,370
Canine parvovirus 2	*Parvoviridae; Protoparvovirus*	1	OZ453693.1	4,775	446
Protoparvovirus Dog/89740/UK/2022 (human–canine-associated parvovirus 1)	*Parvoviridae; Protoparvovirus*	1	OZ453985.1	4,775	180

The most frequently detected viruses were CECoV (13 detections), canine circovirus and canine kobuvirus (5 each), canine bufavirus (4), canine astrovirus (3) and rotavirus C (RVC; 2). Single detections were observed for canine minute virus, canine bocavirus 3, canine parvovirus 2, human–canine-associated parvovirus 1 (HCAPV-1) and sapovirus (Table 1). Per-sample metadata, diagnostic PCR panel results and viral metagenomic detections are provided in Table S1.

## DNA viruses

### Canine parvovirus type 2

**Fig. 3.**
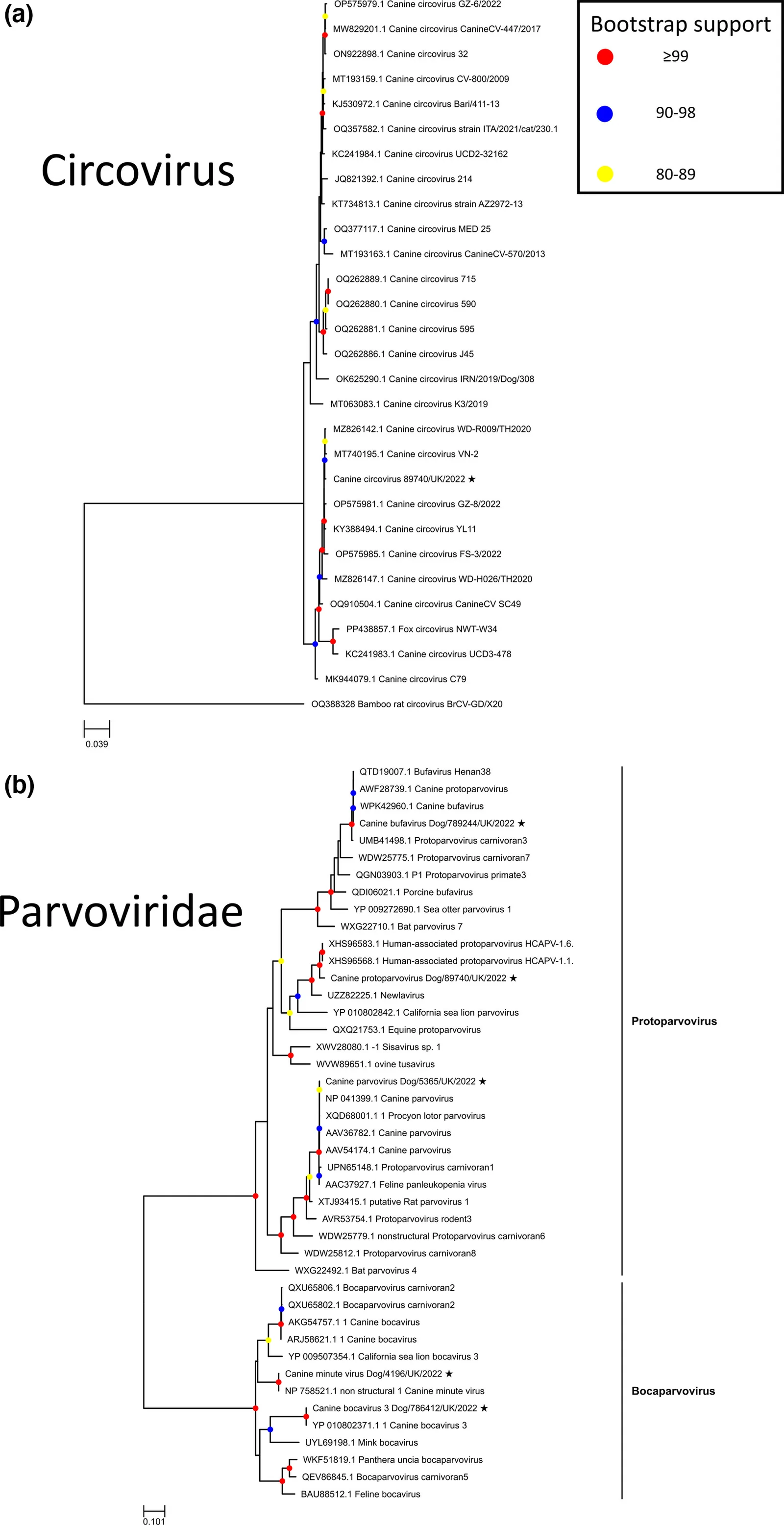
Phylogenetic analysis of ssDNA viruses detected in this study (black stars). For *Circovirus* (**a**), whole genomes were used (nucleotide); for *Parvoviridae* (**b**), NS1 protein sequences were used. The tree includes the nearest database matches and representative ICTV genera. Branch lengths reflect estimated substitutions per site.

**Fig. 4.**
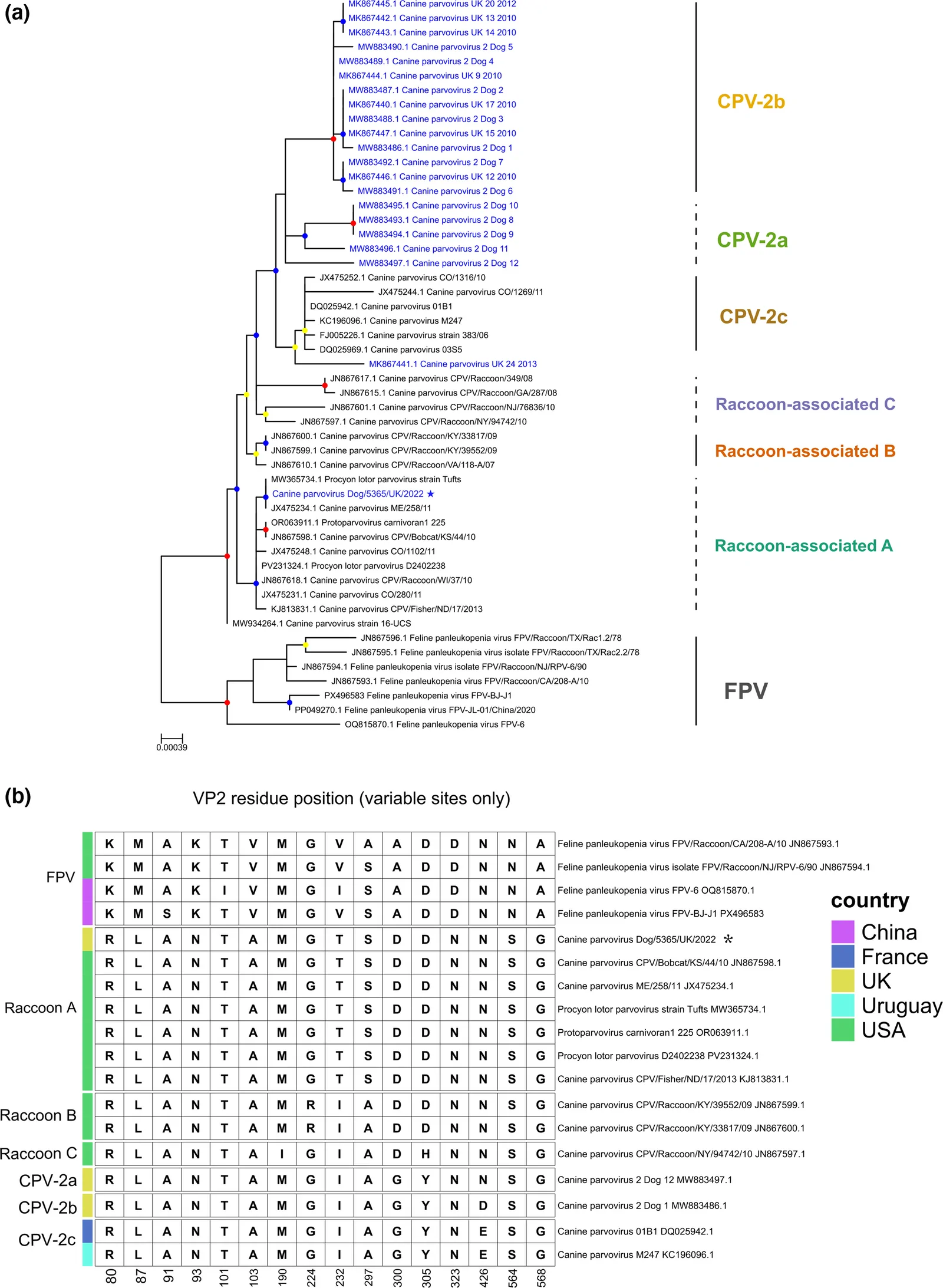
(a) Phylogenetic reconstruction of VP2 capsid protein (1,755 nucleotides). Blue leaves, UK sequences; black stars, this study. (b) Capsid amino acid residues associated with canine parvovirus 2 (CPV-2) lineages isolated from raccoons and bobcat (Raccoon A, **B and **C), which includes the dog sequence from this study (*). Also included are the sequences for feline parvovirus (FPV) and the CPV-2a-, b- and c-related prototypes.

A single canine parvovirus type 2 (CPV-2) genome (CPV-2 Dog/5365/UK/2022) was detected in a sample negative by the diagnostic PCR panel (Fig. 3b). Full-length VP2 phylogenetic analysis placed the strain within one of three basal lineages outside the CPV-2a/2b/2c clade, the predominant domestic dog clade since the 1970s (Fig. 4a). This group is designated here as ‘raccoon-associated’ due to over-representation of *Procyon lotor*–derived sequences, although it is best interpreted more broadly as a wildlife-associated CPV-like lineage, with occasional sequences from other wildlife hosts (such as fisher and bobcat) and a single report from a domestic dog in the USA (OR063911.1). Consistent with this phylogenetic placement, VP2 residue profiling (used for CPV typing) identified 426N at the key discriminatory site that distinguishes CPV-2a/2b/2c variants. However, ancestral residues were retained at 300 and 305 (300D/305D) rather than the canonical CPV-2a/2b/2c background (300G/305Y), consistent with early-diverging CPV genotypes (Fig. 4b). Compiling all UK VP2 CPV-2 sequences, most were placed in CPV-2a or 2b, with CPV-2 Dog/5365/UK/2022 being the first example of this earlier-diverging lineage in the UK and most closely related to *Procyon lotor* parvovirus strain Tufts (MW365734.1), sharing 99.31% nucleotide identity.

### Human–canine-associated parvovirus 1

**Fig. 5.**
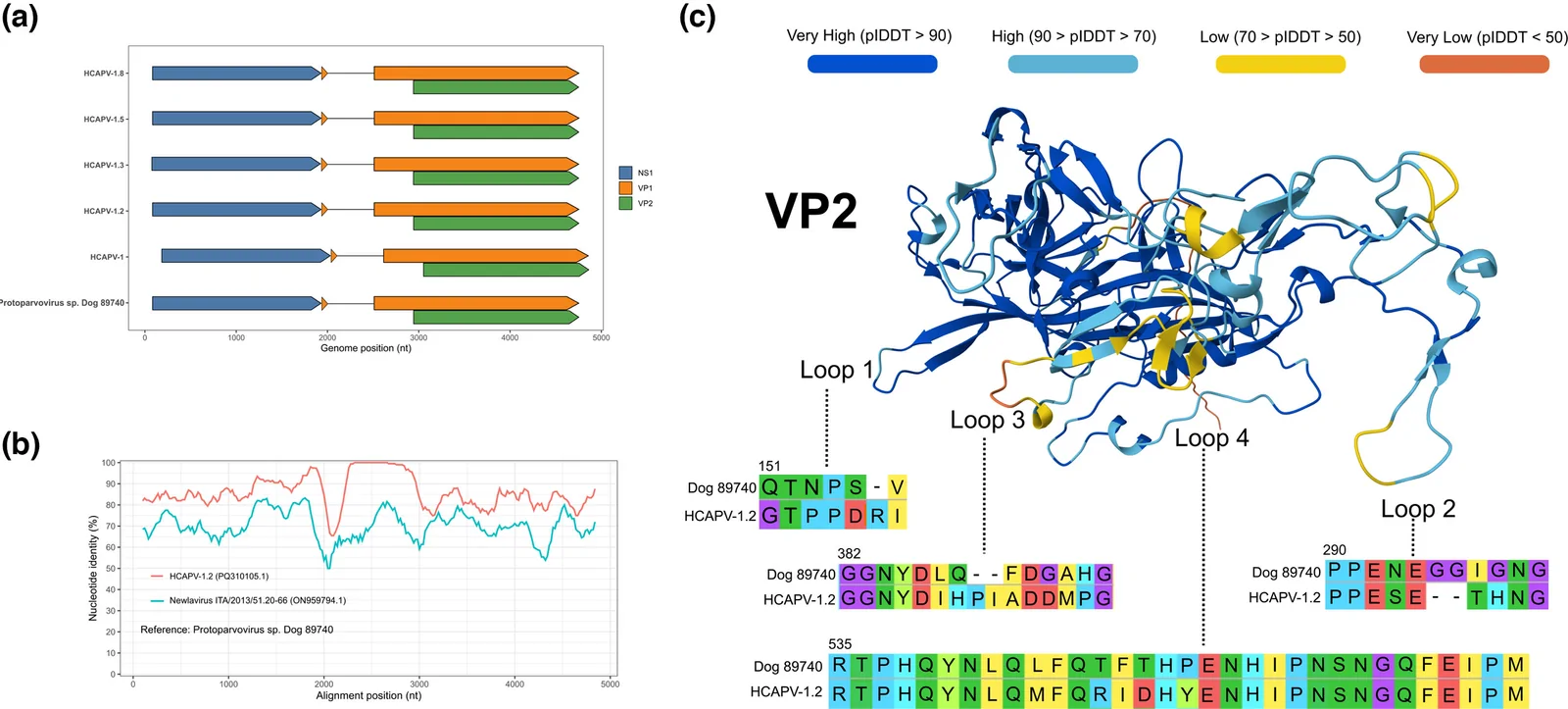
(a) Genome organization for Protoparvovirus Dog/89740/UK/2022 and its closest known relatives, HCAPV-1. (b) Similarity plot analysis comparing nucleotide identity of HCAPV-1.2 and Newlavirus. (c) AlphaFold 3D reconstruction of the VP2 capsid protein for Protoparvovirus Dog/89740/UK/2022. pLDDT, per-residue confidence metric.

Another complete protoparvovirus sp. genome (Protoparvovirus Dog/89740/UK/2022) was recovered from a single individual. Under current ICTV demarcation criteria, this virus falls within the same species as the recently described HCAPV-1 (NS1: 89% aa identity to HCAPV-1.2, PQ310105.1; species threshold: 85%). In NS1 phylogenetic analysis, this species falls as a sister to *Protoparvovirus carnivoran 5*, which encompasses the recently described Newlavirus, a highly prevalent virus of foxes and less commonly dogs (Fig. 3b). The genome architecture of Protoparvovirus Dog/89740/UK/2022 was conserved relative to the closest HCAPV-1-related references (Fig. 5a). Despite species-level relatedness, capsid comparison showed non-uniform divergence. The N-terminal VP1u region was highly similar (~99% amino acid identity) between the UK canine and HCAPV-1 sequences, whereas divergence increased across the VP2-shared capsid core (82% amino acid identity) (Fig. 5b). Alignment-based mapping identified four discrete regions of elevated amino-acid divergence, and structural projection onto the UK VP2-core model showed that these correspond to predicted surface-exposed segments (loops 1–4) (Fig. 5c). In addition, loops 2–4 showed lower pLDDT (predicted Local Distance Difference Test) scores than the VP2 core, a pattern expected for exposed loop regions that are less tightly constrained by local packing interactions than the capsid core.

In addition to Protoparvovirus Dog/89740/UK/2022 and CPV-2 Dog/5365/UK/2022, another protoparvovirus, canine bufavirus, was detected in four dogs. Furthermore, two bocaparvoviruses were also recovered, assigned to canine minute virus (also known as *Carnivore bocaparvovirus* 1; CBoV-1) and canine bocavirus 3 (CBoV-3), based on NS1 phylogenetic placement (Fig. 3b). The CBoV-3 strain (CBoV-3 Dog/786412/UK/2022) shared 98.9% nucleotide identity with the only other publicly available complete genome from a dog in the USA (NC_076995.1). The CBoV-1 strain, on the other hand, grouped with multiple European lineages, with the closest reference (Canine minute virus isolate 4033/Italy/2012; MN947833.1) at 99.3% nucleotide identity.

### Canine circovirus

A complete canine circovirus was detected in dog 89740. Whole-genome phylogenetic analysis placed the strain within the diversity of previously reported canine circoviruses with no evidence of a distinct lineage (Fig. 3a). Read mapping identified the virus in four additional libraries.

## RNA viruses

### Canine enteric coronaviruses

**Fig. 6.**
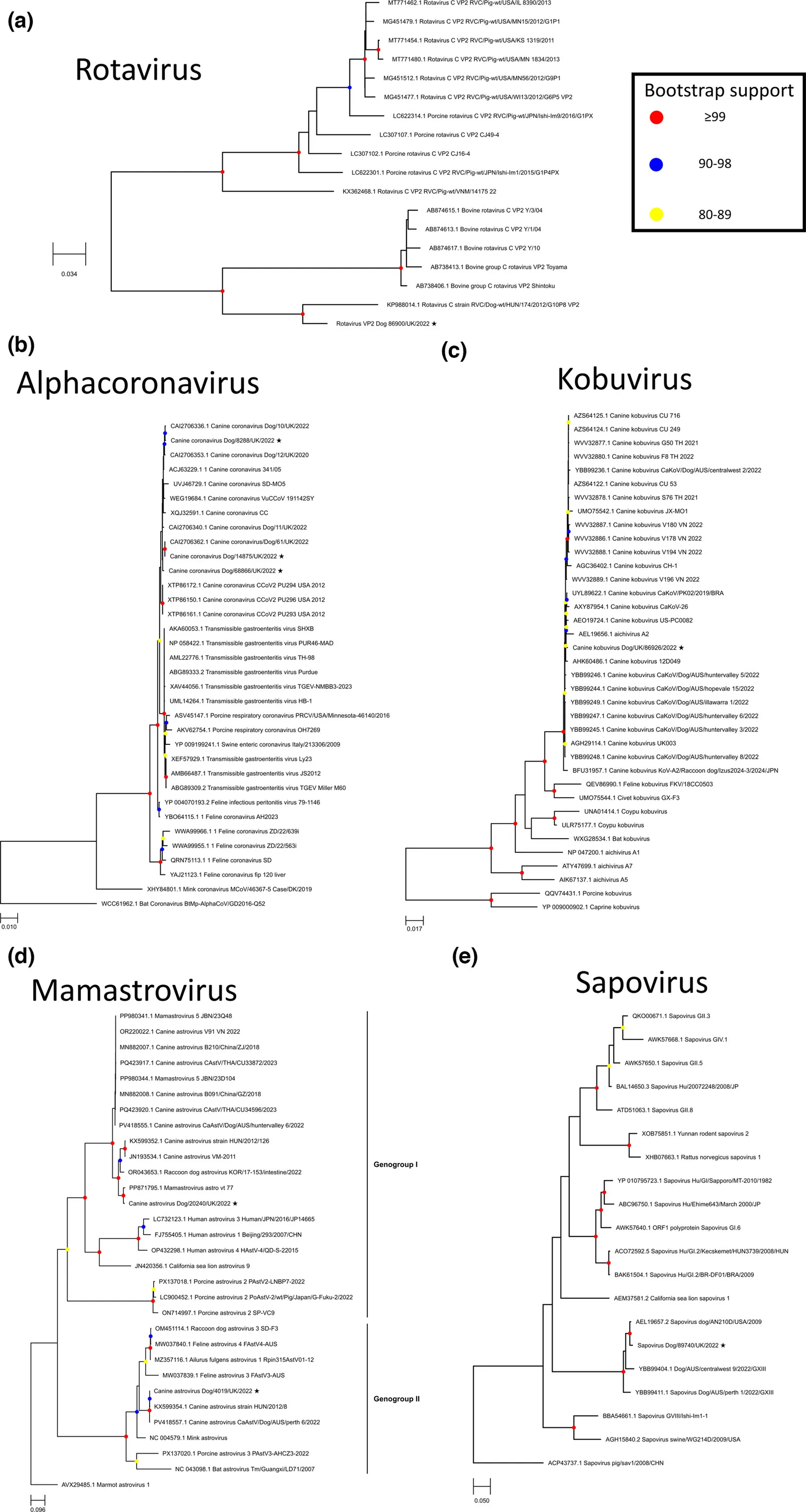
Phylogenetic analysis of ssRNA viruses detected in this study (black stars), based on (a) Rotavirus C (VP2), (b) Alphacoronavirus (RdRp), (c) Kobuvirus (polyprotein), (d) Mamastrovirus (capsid) and (e) Sapovirus (polyprotein). The analysis includes the closest matching sequences and representative members from ICTV-designated genera. Branch lengths reflect evolutionary divergence, measured by the number of amino acid substitutions per site.

Three CECoV genomes were recovered. Comparison with 2022 UK CECoV reference genomes from our previous study [12], which included stool samples collected in the same year, showed that two were near-identical to previously characterized strains (Fig. 6b). Specifically, CECoV Dog 8288/2022 was nearly identical to CECoV Dog 10/2022 (OX335534.1 [12]; >99.9% nucleotide identity), while CECoV Dog 14875/2022 was 100% identical to CECoV Dog 61/2022 (OX335623.1 [12]), previously generated from the same faecal sample [12]. In contrast, the third genome, CECoV Dog 68866/2022, was markedly divergent, with an average nucleotide identity of 96% to its nearest UK-related reference (OX335541.1 [12], Dog 11/2022). Similarity profiling indicated that this divergence was not uniform across the genome, with the strongest signal localized to ORF1a and the nucleocapsid (N) gene, and more modest reductions in the S N-terminal domain–proximal region and the M gene (Fig. S1A). Within ORF1a, the largest drop in identity mapped to nonstructural protein 3 (nsp3), falling below 75% across the macro domain.

### Kobuvirus

A complete canine kobuvirus genome was detected clustering within recognized canine kobuvirus diversity (Fig. 6c). Consistent with the limited variation reported for this group, polyprotein sequences showed low divergence (mean pairwise amino-acid identity=98.5%). This represents a re-detection of canine kobuvirus in the UK since its first report in 2008 [41]. Of the four kobuvirus detections, two were from Scotland (Ayr and Edinburgh) with the other two from South Yorkshire.

### Sapovirus

A partial sapovirus genome of 2,953 nt (of ~7.4 kb) was recovered from the same dog where Protoparvovirus Dog/89740/UK/2022 was also detected. The recovered fragment mapped within the polyprotein region rather than the capsid (VP1). Phylogenetic placement based on this region assigned the virus to genogroup GXIII, a clade comprising viruses reported from canids (Fig. 6e). The nearest available reference sequence was JN387134.2 (USA, 2009), sharing 90.2% nucleotide identity.

### Rotavirus C

RVC was detected in 2 dogs (Dogs 86900 and 4163) from South Yorkshire and Buckinghamshire, with all 11 genome segments recovered for strain characterization from Dog 86900. Segment-based phylogenies placed the UK strain within the canid-associated RVC lineage, clustering with previously described domestic dog strains; where available, related raccoon dog sequences also grouped within this clade (Figs 6a and S2).

### Canine astrovirus

Three dogs were positive for canine astrovirus (Dogs 20240, 20997 and 4019), with two recovered dereplicated genomes showing the canonical astrovirus organization (5′–ORF1a–ORF1b–ORF2–3′). ORF2 (capsid)-based phylogenetic inference placed one strain (20240/UK/2022) in canine astrovirus genogroup I and the other (4019/UK/2022) in genogroup II; genogroup I is predominantly represented by canine sequences, whereas genogroup II is less frequently reported in dogs (Fig. 6d). The highest homology references for the genogroup II strain were from dogs in Hungary and Australia (KX599354.1 and PV418557.1), with 95.3% nucleotide identity to the partial Hungarian genome region and 91.1% to the complete Australian genome. Similarity plot analysis revealed a genome-wide divergence pattern with no discrete ORF2 breakpoint, consistent with background evolutionary distance rather than a capsid-localized event (e.g. recent ORF2 recombination) (Fig. S1B). Sensitivity analyses using relaxed criteria for viral detection identified additional low-level canine astrovirus genogroup I detections. Detections increased from 2 at the primary ≥50% breadth threshold to 7 and 16 at the ≥20% and ≥10% thresholds, respectively (Table S2).

### Comparison with routine diagnostic PCR and high-throughput sequencing co-detections

Comparison between high-throughput sequencing (HTS) and the IDEXX diagnostic PCR was possible only for CECoV and CPV-2, the only two viruses represented in both methods. For CECoV, detection by HTS was significantly associated with PCR positivity (Fisher’s exact test, *P*<0.001), although some discordant samples showed limited HTS mapping support or fell below the breadth threshold used for HTS detection (Supplementary Results; Fig. S3). For CPV-2, cross-platform comparison was limited, with only two discordant observations: one sample was PCR-positive/HTS-negative, and one was HTS-positive/PCR-negative. Within the HTS dataset, multiple viruses were detected in 7 out of 27 virus-positive samples, but no virus–virus co-detection associations remained significant after FDR correction (Supplementary Results; Fig. S3).

## Discussion

Despite advances in HTS, routine veterinary diagnostic uptake remains constrained by cost, workflow complexity and specialist bioinformatic requirements [42]. In this study, HTS broadened virus detection beyond predefined targets and has helped prioritize candidates for further investigation to clarify clinical relevance [7]. Accordingly, from this cohort, we detected 12 viruses using HTS, including several not previously reported in UK dogs with known or suspected enteric pathogenic potential.

### Parvovirus diversity beyond typical domestic dog lineages

CPV-2 is a major cause of acute haemorrhagic gastroenteritis in dogs, as well as a long-standing target of routine vaccination programmes [43]. Following the emergence of the original CPV-2 lineage in the late 1970s, contemporary circulation of strains in domestic dogs has been dominated by CPV-2a/2b/2c lineages [44], which harbour capsid (VP2) substitutions associated with efficient infection and sustained transmission in dogs. A previous large survey of CPV-2 in the UK identified only CPV-2a and -2b [45]. In the present study, we recovered a single divergent CPV-2 strain outside these CPV-2a/2b/2c clades, in a position consistent with prior phylogenies in which wildlife-derived CPV-like viruses branch basal to these domestic dog lineages [46]. In keeping with this, prior experimental work has identified the VP2 300-loop as a major host-range determinant: wildlife-associated CPV variants carrying VP2-300D show reduced infectivity in canine cells, whereas a single 300D→300G substitution can restore infection of canine cells *in vitro* [47]. Accordingly, the presence of VP2-300D in CPV-2 Dog/5365/UK/2022 may provide one plausible explanation for limited establishment in domestic dog populations. However, our detection and a further report from a domestic dog in the USA (OR063911.1) confirm that canine infection by such wildlife-associated variants can nevertheless occur. As a result of these detections in domestic dogs being rare, the clinical relevance of this lineage in pets remains unclear.

This pattern is consistent with occasional spillover into dogs at the domestic–wildlife interface. The absence of raccoons in the UK suggests a broader sylvatic reservoir. Given reports of this clade in non-procyonid carnivores (e.g. mustelids and felids) [48], and evidence for recurrent cross-species transmission among diverse carnivore hosts [46], a priority is coordinated surveillance across domestic and sylvatic animals. In the UK, mustelids such as badgers, pine martens and otters represent practical initial targets for such efforts.

A further important observation was the recovery of a protoparvovirus (Protoparvovirus Dog/89740/UK/2022) that meets ICTV species criteria [49] for HCAPV-1. HCAPV-1 was first reported in China from human oropharyngeal secretions, with additional detections in canine tissues and oropharyngeal swabs [50]. However, in the subset of pet dogs and their close human contacts, HCAPV-1-positive humans and dogs were not identified within the same matched human–dog pairs, limiting inference about direct transmission. Genomic comparison of Protoparvovirus Dog/89740/UK/2022 with available HCAPV-1 sequences suggests that capsid divergence is concentrated in predicted surface-exposed regions, focusing attention on these sites as candidates for potential effects on host interaction and immune recognition. Notably, we detected this virus in faeces, whereas the initial report was based primarily on oropharyngeal secretions and lymph node tissue. Accordingly, tissue tropism cannot be inferred, and faecal detection could reflect enteric replication, systemic shedding or transient passage. Priority follow-up should focus on case-control studies in dogs to evaluate disease association, alongside *in vitro* studies assessing potential host range using cell culture systems.

### Detection of an additional CECoV lineage during the 2022 UK outbreak

Another common virus, CECoV, was detected in several of the dogs sampled. These genomes were obtained from samples collected in 2022 when contemporaneous genomic analyses identified circulation of a CECoV lineage closely related to the virus associated with a 2020 UK vomiting outbreak but carrying additional spike-gene recombination [12, 51]. Within our dataset, one genome corresponded to this recombinant 2022 lineage, one clustered with the clade containing the 2020 minor variant, and a third fell within this minor-variant clade but showed additional divergence concentrated in ORF1a (including nsp3). Variation in the nsp3 macrodomain, a region associated with ADP-ribosylation–mediated antiviral responses [52], underscores the value of whole-genome surveillance for identifying emergent variants that may have distinct viral fitness impacts and may be missed by single-locus typing. This complements our earlier outbreak work showing that reliance on single-gene sequencing alone can fail to resolve key genome-wide differences, including recombination signals that are only apparent from whole-genome comparisons [12].

### Reference gaps and complex infection dynamics

Further detections in this study highlight that publicly available reference genomes for canine enteric viruses remain uneven, with several taxa and lineages still underrepresented. Viral metagenomic screening therefore has particular value for establishing baseline circulation and identifying candidates for prioritized follow-up. More broadly, the capacity to characterize co-detections supports a shift away from strict one-pathogen/one-syndrome interpretations of canine enteric disease, where viral burden, host factors and coinfections jointly shape clinical expression [3, 5, 53, 54]. In that regard, detections of viruses with potential immunomodulatory relevance warrant consideration as contextual modifiers in future studies. For example, it has been suggested that canine circovirus–CPV-2 co-detections, like those detected in this study, have been proposed to enhance CPV-2 replication by promoting immunosuppression, potentially via canine circovirus antagonism of type I IFN (IFN-I) responses [55].

### Interpreting UK-first detections in dogs

Our data provide several examples of viruses that may be overlooked by current surveillance strategies. Although RVC has previously been reported in humans in the UK [56], we report the first detections locally of canine sapovirus, canine astrovirus genogroup II, canine bufavirus, canine minute virus and canine bocavirus 3 (Table S4). Several of these detections justify targeted follow-up because there are prior signals of clinical relevance. For example, canine bocavirus 3 has been detected previously in the liver of a dog following severe illness and death, supporting pathogenic potential [57]. Similarly, canine sapovirus has been detected in dogs with acute gastroenteritis but not in healthy controls, supporting a possible association with clinical disease [58]. Furthermore, the first UK detection of the known pathogen canine minute virus (CnMV/CPV-1) [59] indicates the extent to which internationally recognized canine viruses may be missed locally, supporting periodic, HTS-guided updating of PCR targets.

Other viruses warrant follow-up because sparse genomic representation in dogs likely reflects limited investigation and/or poor detectability with existing panels. For instance, the RVC detection in this study is consistent with the presence of a canid-associated clade [60] that remains poorly represented in available sequence data, despite extensive molecular characterization of RVC in pigs and humans and, to a lesser extent, cattle [61]. Similarly, although sapoviruses are most extensively characterized in humans and pigs [62, 63], our partial genome adds to the limited sequence data available for the canid-associated genogroup GXIII lineage [9, 64].

Despite these findings, virus detection alone does not establish causation in individual diarrhoeal cases [65]. Accordingly, this study primarily informs virus circulation and genomic diversity, not attributable risk. For several taxa identified here (including kobuvirus and bufavirus), published epidemiological evidence for disease association remains unclear [41, 58, 66], and interpretation in the present study is further constrained by the absence of matched healthy controls. Nevertheless, these data provide a useful snapshot of viruses detected in UK clinical submissions. From a diagnostic perspective, these findings help prioritize additional targets for evaluation and possible inclusion in testing workflows. However, as economic and operational barriers continue to decline, HTS may become increasingly feasible for routine veterinary surveillance and, in some settings, shift from an adjunct to a first-line detection approach [42].

## Conclusion

In summary, we have expanded the known circulating viral diversity of UK domestic dogs, including seven taxa not previously reported in UK dogs. While detection alone cannot establish disease causation, these findings indicate that routine targeted testing may under-represent circulating enteric viral diversity and support an adaptive diagnostic approach that combines PCR screening with periodic genome-informed recalibration, helping to define priorities for targeted epidemiological and functional follow-up.

## Declaration of generative AI use

During the preparation of this manuscript, J.P. used GPT-5.4 (OpenAI) to assist with language refinement and sentence restructuring. After using this tool, the authors reviewed and edited the content as needed and take full responsibility for the content of the published article.

## Supplementary material

10.1099/mgen.0.001799 Supplementary Material 1.

10.1099/mgen.0.001799 Supplementary Material 2.

## Abbreviations

CDPP: Canine Diarrhoea PCR Panel
CECoV: canine enteric coronavirus
CPV-2: canine parvovirus type 2
ENA: European Nucleotide Archive
HCAPV-1: human–canine-associated parvovirus 1
HTS: high-throughput sequencing
ICTV: International Committee on Taxonomy of Viruses
IFN-I: type I IFN
nsp3: nonstructural protein 3
pLDDT: predicted Local Distance Difference Test
RVC: rotavirus C
SISPA: sequence-independent single-primer amplification
