# Supplementary material for: Expanded detection of canine enteric viruses in UK dogs with diarrhoea
Source: Microb Genom. 2026 Sep 8;12(9):001799. doi: 10.1099/mgen.0.001799 (PMC13552968; doi:10.1099/mgen.0.001799)
Supplement: Supplementary file 1 [file mgen-12-01799-s001.pdf]

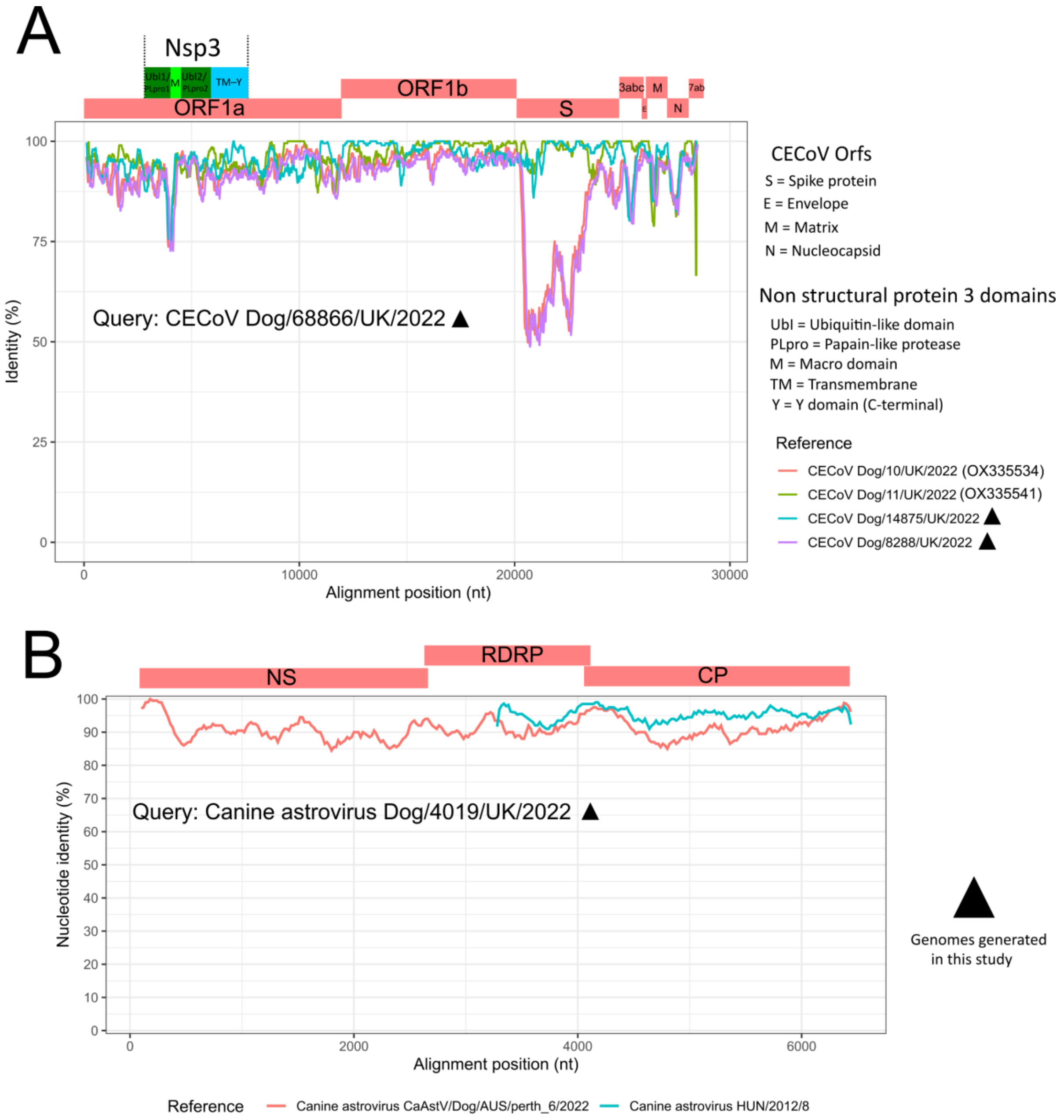

Figure S1. Similarity plot analyses for canine enteric coronaviruses (**A**) and canine astroviruses genogroup II (**B**) found in this study.

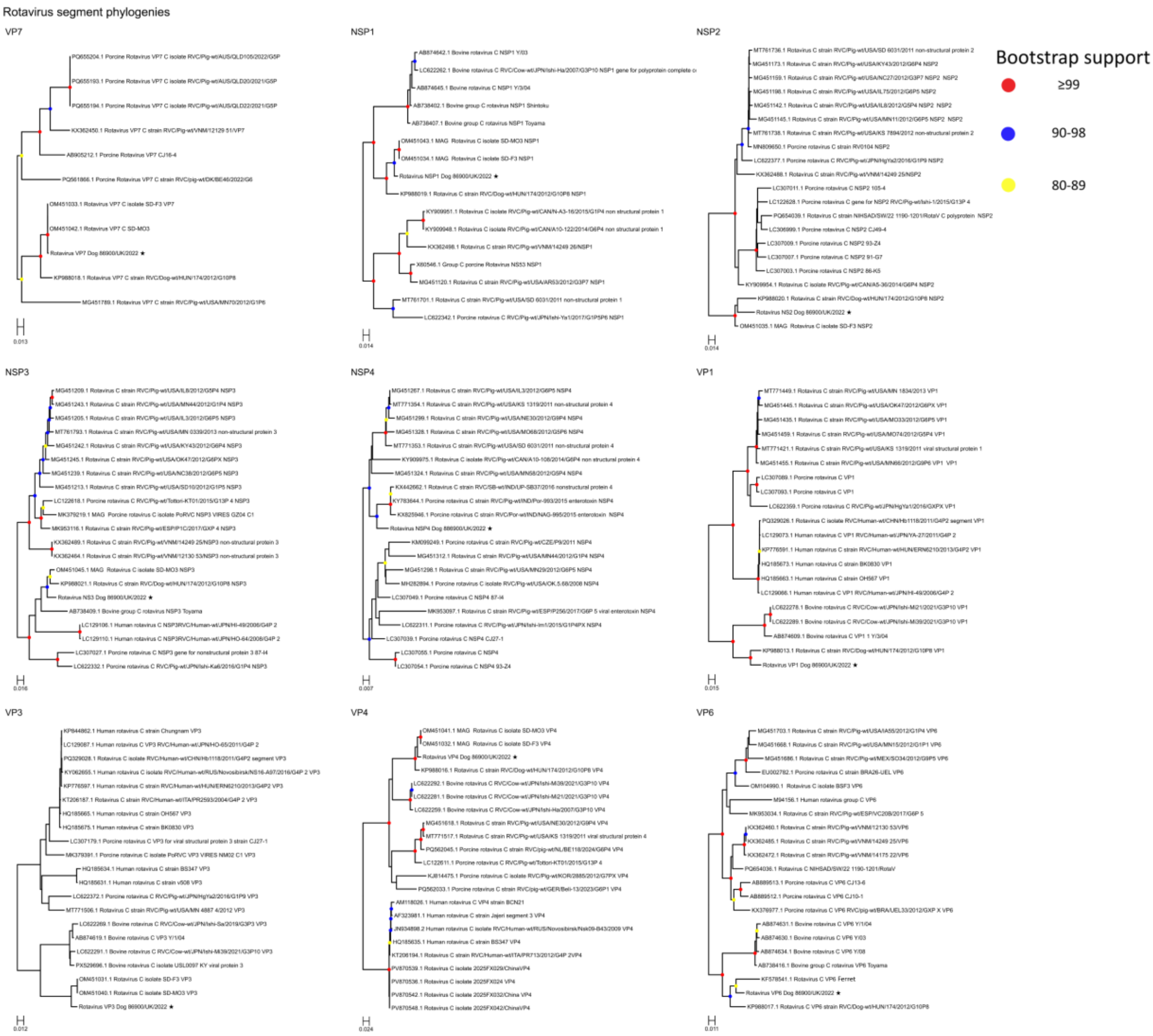

Figure S2. Phylogenetic analysis of single-stranded Rotavirus C segments detected from Dog 86900 (black stars). The analysis includes the closest matching sequences. Branch lengths reflect evolutionary divergence, measured by the number of nucleotide substitutions per site.

Supplementary Results: Comparison of HTS detections with routine diagnostic PCR and HTS co-detections

Comparison with routine diagnostic PCR was possible only for canine enteric coronavirus (CECoV) and canine parvovirus-2 (CPV-2), the two viruses represented on the diagnostic PCR panel. For CECoV, among the 68 samples with corresponding CECoV PCR data, 7 were positive by both HTS and PCR, 3 were HTS-positive/PCR-negative, 5 were HTS-negative/PCR-positive, and 53 were negative by both methods. Under the HTS calling criteria, detections required at least 50% read-mapping breadth across the corresponding viral contig; consequently, samples classified as HTS-negative could still show sub-threshold mapping support. These comparisons should therefore be interpreted cautiously, as they were limited to two viruses and were influenced by analytical sensitivity and HTS detection thresholds.

Relative to concordant CECoV-positive samples (HTS+/PCR+), discordant HTS+/PCR− samples had lower CECoV mapping support, with reduced median mapped read count (5,516 vs 15,437) and median reads per million (25,354 vs 54,913). Among PCR+/HTS− samples, two showed near-threshold mapping breadth (41.62–42.33%; 186–193 mapped reads), whereas the remaining three showed little or no detectable mapping support (0–0.76% breadth; 0–2 mapped reads).

Across the full dataset, CECoV was the only virus showing a significant association between HTS and PCR detection after FDR correction (Supplementary Figure S3A). For CPV-2, comparison between methods was limited to two discordant observations: one sample was PCR-positive/HTS-negative, and one early-diverging CPV-2 lineage detected by HTS was not detected by the diagnostic PCR panel. Within the HTS dataset, multiple viruses were detected in 7 of 28 virus-positive samples, but no virus–virus co-detection associations remained significant after FDR correction (Supplementary Figure S3B).

<u>Contingency table comparing HTS and routine diagnostic PCR results for CECoV and CPV-2</u>

Virus	HTS+/PCR+	HTS+/PCR−	HTS−/PCR+	HTS−/PCR−
CECoV	7	3	5	53
CPV-2	0	1	1	72

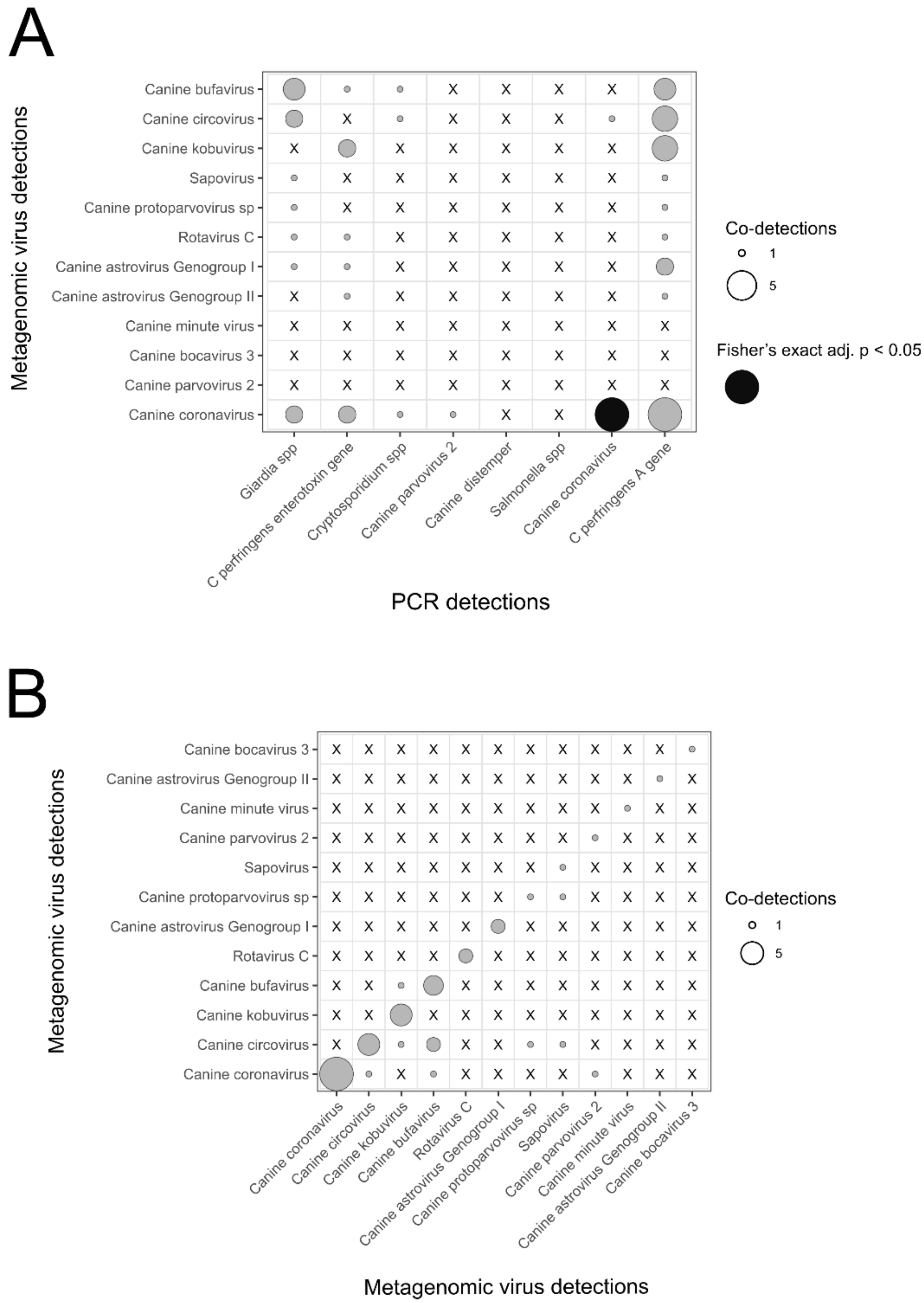

Figure S3. Bubble matrices summarising co-detections across libraries: **A)** metagenomic viruses versus PCR targets, and **B)** metagenomic viruses versus metagenomic viruses. Bubble size indicates the number of libraries in which both features were detected (larger bubbles = more co-detections); crosses indicate no co-detections.

Supplementary Code S1. R script for sliding-window nucleotide identity analysis

This R script calculates sliding-window nucleotide identity between a query sequence and selected comparator sequences from a MAFFT-aligned nucleotide FASTA file. Identity was calculated using a 200 nt window and 20 nt step, excluding positions containing gaps or ambiguous bases in either sequence. The script outputs per-window identity values and corresponding plots.

Software requirements

R version >4.0

```
# ============================================================

# Minimal sliding-window nucleotide identity plot (SimPlot-style)

# - Input: MAFFT-aligned multi-FASTA (nucleotide)

# - Output: TSV of window identities + PNG/PDF/SVG plot

# - Identity: computed over positions where BOTH sequences are non-gap and non-N

# ============================================================


suppressPackageStartupMessages({

  library(seqinr)

  library(ggplot2)

})


# -------------------------

# User inputs (edit these)

# -------------------------

aln_fasta <- "D:/path/to/aligned_sequences.fas"   # MAFFT-aligned nucleotide FASTA

query_header <- "QUERY_NAME_EXACTLY_AS_IN_FASTA"
```

```
# Provide a named vector: names are legend labels, values are exact FASTA headers
comparators <- c(
  "Comparator 1" = "COMPARATOR1_HEADER_EXACT",
  "Comparator 2" = "COMPARATOR2_HEADER_EXACT"
)

out_prefix <- "D:/path/to/output/simplot_like"    # writes .tsv .png .pdf .svg .log
window_size <- 200
step_size   <- 20

# ------------------------
# Helpers
# ------------------------
msg <- function(...) cat(sprintf(...), "\n")

read_aln <- function(f) {
  if (!file.exists(f)) stop("Alignment FASTA not found: ", f)
  read.fasta(file = f, seqtype = "DNA", as.string = FALSE,
             forceDNAtolower = FALSE, whole.header = TRUE)
}

get_seq <- function(aln, header) {
  nm <- trimws(names(aln))
  idx <- which(nm == header)
```

```
  if (length(idx) == 0) {
    stop("Header not found in FASTA: ", header, "\nAvailable headers include:\n  - ",
         paste(head(nm, 10), collapse = "\n  - "))
  }
  toupper(aln[[idx[1]]])
}

sliding_identity <- function(q, c, window, step) {
  L <- length(q)
  if (L != length(c)) stop("Aligned sequences differ in length.")
  if (L < window) stop("Window size > alignment length.")

  starts <- seq(1, L - window + 1, by = step)
  out <- lapply(starts, function(s) {
    e <- s + window - 1
    qwin <- q[s:e]
    cwin <- c[s:e]

    valid <- qwin != "-" & cwin != "-" & qwin != "N" & cwin != "N"
    denom <- sum(valid)
    pid <- if (denom == 0) NA_real_ else 100 * sum(qwin[valid] == cwin[valid]) / denom

    data.frame(start = s, end = e, mid = floor((s + e)/2),
               identity = pid, n_valid = denom)
```

```
  })
  do.call(rbind, out)
}

# -------------------------
# Run
# -------------------------
log_file <- paste0(out_prefix, ".log")
sink(log_file, split = TRUE)
on.exit(sink(), add = TRUE)

msg("R: %s", R.version.string)
msg("Input alignment: %s", aln_fasta)
msg("Window=%d nt; Step=%d nt", window_size, step_size)

aln <- read_aln(aln_fasta)
msg("Sequences in alignment: %d", length(aln))
msg("First headers:\n%s", paste(head(trimws(names(aln)), 10), collapse = "\n"))

q <- get_seq(aln, query_header)

# Calculate per comparator
all_df <- list()
for (lab in names(comparators)) {
```

```
  hdr <- comparators[[lab]]

  cseq <- get_seq(aln, hdr)

  df <- sliding_identity(q, cseq, window_size, step_size)

  df$comparator <- lab

  all_df[[lab]] <- df

  msg("Comparator '%s': median identity = %.2f (n_valid median=%d)",

      lab, median(df$identity, na.rm = TRUE), as.integer(median(df$n_valid, na.rm = TRUE)))

}


df_all <- do.call(rbind, all_df)


# Save TSV

tsv_file <- paste0(out_prefix, ".tsv")

write.table(df_all, file = tsv_file, sep = "\t", quote = FALSE, row.names = FALSE)

msg("Wrote TSV: %s", tsv_file)


# Plot

p <- ggplot(df_all, aes(x = mid, y = identity, color = comparator)) +

  geom_line(linewidth = 0.9, na.rm = TRUE) +

  scale_y_continuous(limits = c(0, 100), breaks = seq(0, 100, 10),

                     expand = expansion(mult = c(0.01, 0.01))) +

  labs(x = "Alignment position (nt)",

       y = "Nucleotide identity (%)",

       color = paste0("Comparator (query: ", query_header, ")")) +
```

```
  theme_bw(base_size = 12) +
  theme(legend.position = "top")

png_file <- paste0(out_prefix, ".png")
pdf_file <- paste0(out_prefix, ".pdf")
svg_file <- paste0(out_prefix, ".svg")

ggsave(png_file, p, width = 8.5, height = 4.8, dpi = 600)
ggsave(pdf_file, p, width = 8.5, height = 4.8)
ggsave(svg_file, p, width = 8.5, height = 4.8, device = "svg")

msg("Wrote PNG: %s", png_file)
msg("Wrote PDF: %s", pdf_file)
msg("Wrote SVG: %s", svg_file)
msg("Log: %s", log_file)

print(p)
```
